# Prognostic implication of right ventricular-pulmonary artery coupling in valvular heart disease

**DOI:** 10.3389/fcvm.2024.1504063

**Published:** 2025-01-14

**Authors:** Zhenni Wu, Mingxing Xie, Li Zhang, Qing He, Lang Gao, Mengmeng Ji, Yixia Lin, Yuman Li

**Affiliations:** ^1^ Department of Ultrasound Medicine, Union Hospital, Tongji Medical College, Huazhong University of Science and Technology, Wuhan, China; ^2^ Clinical Research Center for Medical Imaging in Hubei Province, Wuhan, China; ^3^ Hubei Province Key Laboratory of Molecular Imaging, Wuhan, China

**Keywords:** right ventricular-pulmonary artery coupling, valvular heart disease, pulmonary hypertension, congenital heart defect, right ventricular function, clinical utility

## Abstract

Valvular heart disease (VHD) leading to inadequate hemodynamic circulation is a major cause of cardiovascular morbidity and mortality worldwide. Right ventricular-pulmonary artery (RV–PA) coupling integrates the ability of RV contractility to adapt to increased pulmonary arterial afterload. If the right ventricle cannot adapt to the elevated afterload by increasing its contractile function, RV-PA uncoupling occurs. RV-PA uncoupling has been shown to be associated with poor outcomes in VHD. This review summarizes the prognostic significance of RV-PA coupling in patients with VHD.

## 1 Introduction

Valvular heart disease (VHD), including congenital and acquired valvular diseases, is a major cause of cardiovascular morbidity and mortality worldwide, affecting more than 2% of the population (1). Previous studies have demonstrated the impact of right ventricular (RV) dysfunction on the poor prognosis in patients with VHD (2, 3). In recent years, right ventricular-pulmonary artery (RV-PA) uncoupling has emerged as a strong predictor of poor prognosis in VHD (4–6). RV-PA coupling integrates the ability of the right ventricle to maintain adequate output in the face of increased pulmonary arterial afterload. When the RV-PA coupling is normal, the right ventricle can adapt to an elevated afterload by increasing its contractile function, and the ratio may remain within the normal range (7–9). However, with prolonged elevation of RV afterload, RV remodeling occurs, leading to a progressive impairment of RV systolic function (8–10). This inadequacy compromises the maintenance of normal cardiac output, leading to RV uncoupling from the PA and ultimately to RV dysfunction and RV failure (11, 12). Moreover, RV-PA uncoupling indicates an advanced stage of disease progression (5, 13). Therefore, identification of such high-risk patients in clinical practice is of paramount importance for clinical decision making. With the increasing focus on the measurement of RV-PA coupling in VHD patients, a growing number of studies have demonstrated its strong impact on prognosis. In this review, we focus on the prognostic significance of RV-PA coupling in patients with VHD.

## 2 Assessment of right ventricular-pulmonary arterial coupling

### 2.1 Invasive method

**Figure 1:**
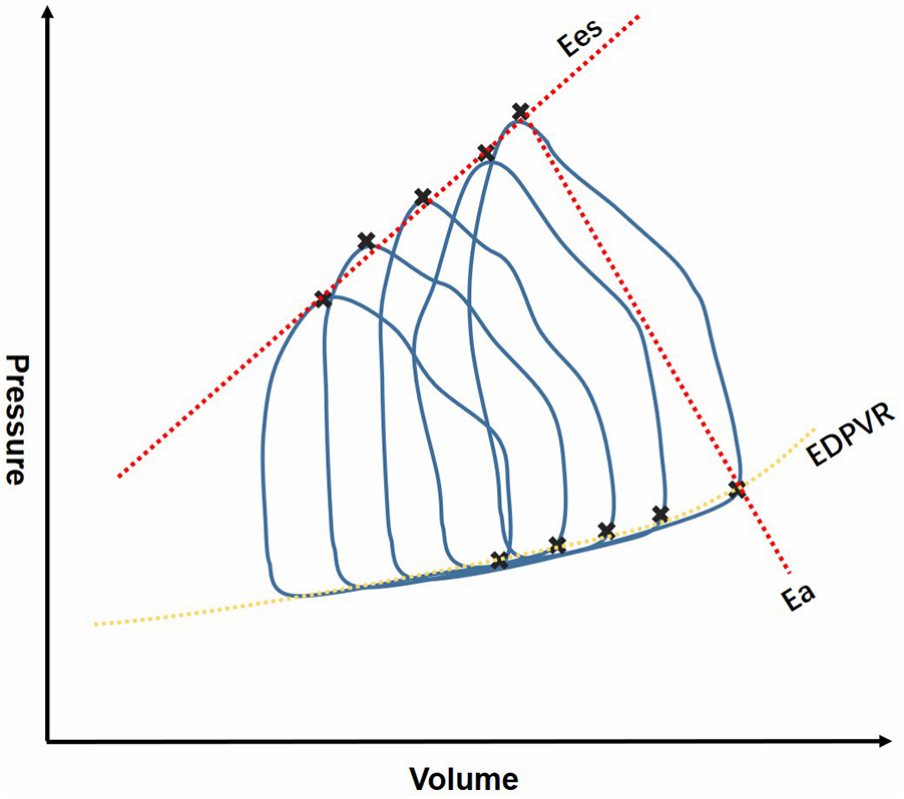
Invasive measurement of RV-PA coupling derived from RHC. RHC, right heart catheterization; Ees, end-systolic elastance; Ea, elastance; EDPVR, end systole pulmonary vascular resistance.

RV-PA coupling has gained more attention in various clinical practice, such as heart failure, pulmonary hypertension, VHD and congenital heart defect (CHD) (14–16). RV-PA coupling can be assessed by invasive and noninvasive metrics, and the gold standard for evaluating RV-PA coupling is the ratio of single-beat and multi-beat RV end-systolic elastance (Ees) to PA elastance (Ea) derived from invasive pressure-volume loops by right heart catheterization(RHC) (7, 9, 17–19). Measurement of Ees/Ea is shown in Figure 1. An Ees/Ea ratio between 1.5 and 2.0 is considered to be normal range according to previous studies (20), indicating that RV contractility adapts to the afterload with the highest efficiency (21, 22). Some of the studies use Ees/Ea to represent RV-PA coupling, and the most favorable RV-PA coupling is acquired when Ees/Ea = 1 (23). This method is considered to be more sensitive and accurate, however, it is difficult to apply conventionally due to its high cost, technical skill requirements and critical conducting condition (24). Additionally, researchers have found that Ees and Ea may be abnormal, but the ratio remains within the normal range. Thus, non-invasive alternative indicators have been introduced to meet the demand for clinical use.

### 2.2 Non-invasive methods

Cardiac magnetic resonance (CMR) imaging has been considered the gold standard for the non-invasive assessment of RV function and RV-PA coupling (25). The ratio of CMR-derived RV end-systolic volume (ESV) to stroke volume (SV) has been shown to correlate with cardiac catheterization-derived RV-PA coupling (26).

Currently, the most commonly used echocardiographic surrogate in daily practice is the ratio of tricuspid annular plane systolic excursion (TAPSE) obtained from M-mode imaging in the apical RV-focused four-chamber view to pulmonary arterial systolic pressure (PASP) derived from RV systolic pressure (RVSP) (9, 10, 19, 27). PASP is calculated from peak tricuspid regurgitant velocity using the Bernoulli equation summing right atrial pressure. TAPSE and PASP represent RV contraction and RV afterload, separately. However, TAPSE tends to decrease after cardiac surgery even when overall RV function remains normal (28), which may underestimate RV-PA coupling.

**Figure 2:**
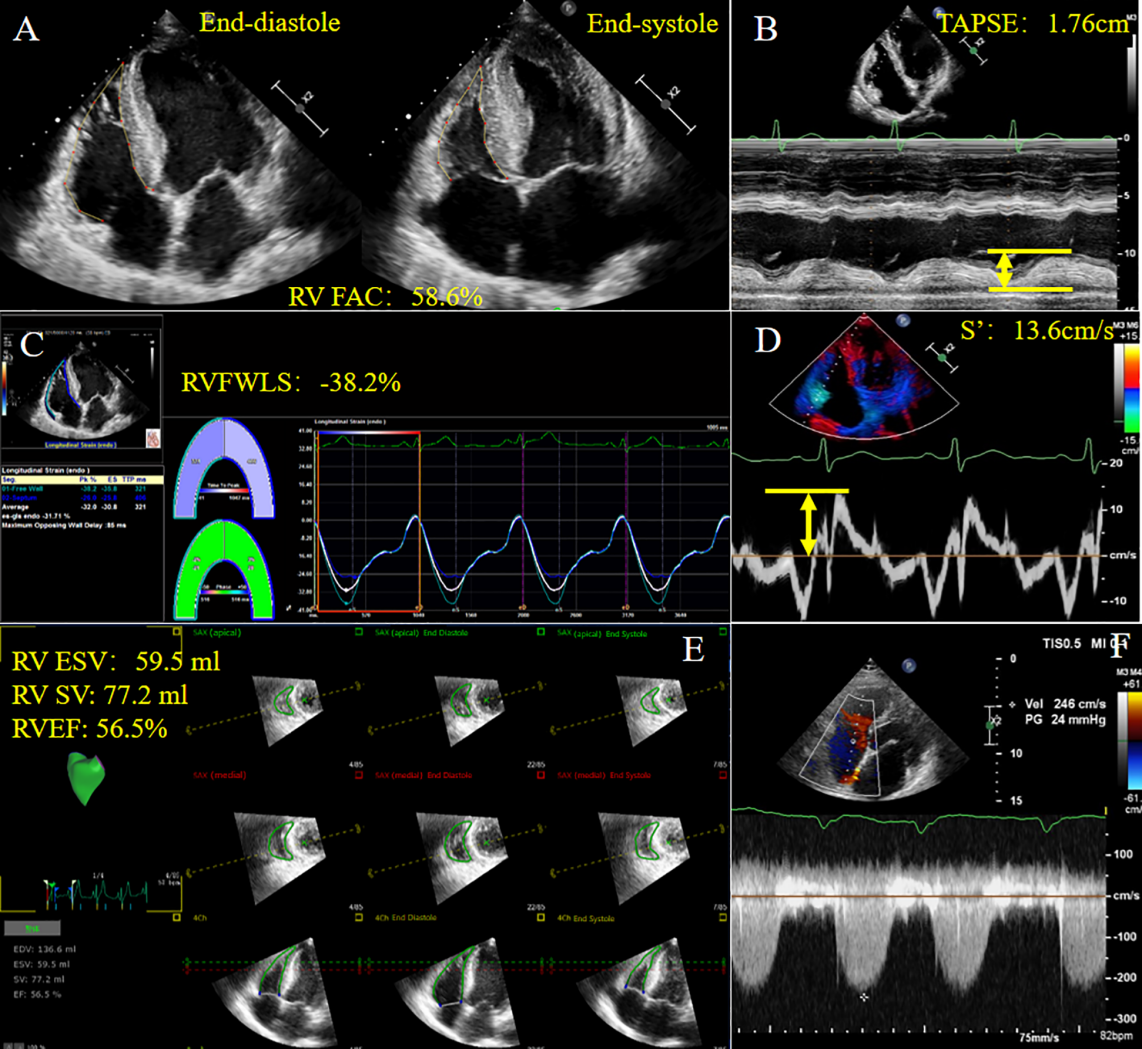
Non-invasive measurement of RV function and RV-PA coupling parameters derived from echocardiography in a patient with HFpEF. **(A)** RVFAC; **(B)** TAPSE; **(C)** RVFWLS; **(D)** S′; **(E)** 3D-TTE derived RV ESV, RV SV, RVEF; **(F)** Tricuspid regurgitation spectrum. HFpEF, heart failure with preserved ejection fraction; RVFAC, right ventricular fractional area change; TAPSE, tricuspid annular plane systolic excursion; RVFWLS, right ventricular free wall longitudinal strain; S′, tricuspid annular systolic velocity; ESV, end systolic volume; SV, stroke volume; RVEF, right ventricular ejection fraction. Yellow arrows represent measurements for parameters.

Novel methods to account for RV-PA coupling, such as three-dimensional echocardiography (3DE) and RV strain, are gaining increasing attention. Acquisition of ESV/SV by 3DE using offline software has been shown to be associated with invasively measured RV-PA coupling from cardiac catheterization in patients with pulmonary hypertension (29). 3DE can be performed postoperatively to avoid assessment of TAPSE and RV geometric hypothesis. The ratio of RV free wall longitudinal strain (RVFWLS) obtained by speckle-tracking echocardiography (STE) to PASP has also been used in clinical practice (30), and has shown its potential value in pulmonary hypertension patients compared with conventional echocardiographic parameters (9). Apart from the above-mentioned indices, the ratios between fractional area change (FAC), right ventricular ejection fraction (RVEF) or tricuspid annular peak systolic velocity (S′) measured by tissue Doppler imaging and PASP or RVSP are less commonly used (13, 31). Non-invasive measurement of RV function and RV-PA coupling parameters are shown in Figure 2.

## 3 Acquired valvular diseases

Acquired valvular diseases refer to situations that develop after birth due to non-congenital factors. These diseases can result from a range of causes, including degenerative processes, infections, inflammatory conditions, and other acquired heart diseases (32). These acquired valvular diseases can significantly impact cardiac function and require medical or surgical intervention, including the use of medications, valve repair, or valve replacement (33). The management of these conditions often involves a multidisciplinary approach, taking into account the patient's overall health and the specific valve involved. In this review, we choose three main categories that have been discussed more often to represent acquired valvular diseases.

### 3.1 Aortic valve

Severe aortic stenosis (AS) results in inadequate output, leading to left ventricular (LV) remodeling, heart failure (HF) or even death (34). In patients with severe AS, reduced LV output leads to chronic pressure overload in the LV chambers, retrograde transmission of the pressure through the pulmonary vasculature, and results in compensatory RV and pulmonary vasculature remodeling, RV dilatation or RV dysfunction (35, 36). A previous study has also revealed that RV dysfunction is common in AS patients due to RV-LV interdependence (37). Both RV dysfunction and pulmonary hypertension have been shown to be associated with worse outcomes in patients undergoing aortic valve implantation (38, 39), confirming the fundamental role of preoperative assessment of RV function. RV-PA coupling as a novel marker of RV function and pulmonary hypertension has demonstrated its prognostic value in postoperative patients.

#### 3.1.1 Transcatheter aortic valve replacement

Transcatheter aortic valve replacement (TAVR) is a less invasive approach for patients with severe AS requiring intervention, associated with a milder recovery and shorter hospital stay (40, 41). However, mortality varies from 19.3%–67.8% during 2–5 years of postoperative follow-up, requiring sensitive and detailed assessment of the factors associated with mortality and outcome (42, 43). Thus, the predictive value of baseline RV-PA coupling in TAVR candidates with severe symptomatic AS has gained significant interest. The impact of RV-PA coupling after TAVR is shown in Table 1.

**Table 1:** Studies examining the utility of RV-PA coupling among SAVR or TAVR recipients.

Author, year	Modality	Study population	RV-PA uncoupling cutoff	Follow up	Baseline RV-PA Uncoupling^a^	Main results
Sultan et al. (5)	TTE	457 patients underwent TAVR from a single U.S. center	<0.59 mm/mmHg	2 years	74.2%	TAPSE/PASP ratio is significantly associated with all-cause mortality in TAVR recipients as a continuous variable
Eleid et al. (44)	TTE	44 patients underwent TAVR from a single U.S. center	Not identified	30 days	-	TAVR significantly improved RV-PA coupling, represented by S′/RVSP ratio. The ratio can be used to assess the efficacy of TAVR and to monitor patient status.
Vizzardi et al. (45)	TTE	56 patients underwent TAVR from a single Italian center	Not identified	10 years	-	TAPSE/PASP and RVLS/PASP ratios are independently associated with the composite of death and hospitalization for HF
Adamo et al. (46)	TTE	377 patients underwent TAVR in the Italian ClinicalService CoreValve Project	<0.36 mm/mmHg	6 months	19%	TAPSE/PASP ratio is strongly associated with all-cause mortality irrelevant of surgical risk score
Cahill et al. (6)	TTE	570 low-risk patients with severe AS in the PARTNER 3 trial underwent TAVR or SAVR	<0.55 mm/mmHg	2 years	38.9%	Baseline RV-PA uncoupling is independently associated with all-cause mortality, cardiovascular mortality, and rehospitalization
Parasca et al. (47)	TTE	132 patients underwent TAVR from a single Romanian center	<0.63%/mmHg^b^	2.47 years (mean data)	50.8%	Impaired RVFWLS/PASP ratio is strongly associated with high death rate and rehospitalization
Alwan et al. (48)	TTE	404 patients underwent TAVR from a single Swiss center	<0.39 mm/mmHg	1 year	43.1%	Post-TAVR patients with RV-PA uncoupling is not statistically associated with all-cause mortality, but showed a trend towards a higher risk of cardiac mortality
Meucci et al. (36)	TTE	900 patients underwent TAVR in 2 European tertiary centers	<0.55 mm/mmHg	40 months (median data)	58%	Pre-TAVR TAPSE/PASP ratio is not associated with long-term mortality. Post-TAVR TAPSE/PASP ratio is independently associated with long-term mortality
Hakgor et al. (49)	TTE	403 patients underwent TAVR from a single tertiary center	<0.55 mm/mmHg	2 years	70.2%	Reduced preoperative TAPSE/PASP ratio is associated with in-hospital and 2-year all-cause mortality.
Silva et al. (50)	TTE	985 AS patients from the PARTNER 2A randomized trial and 719 patients from the SAPIEN 3 intermediate-risk tertiary.	<0.55 mm/mmHg	1 year	-	TAPSE/PASP ratio improved following TAVR but deteriorated significantly following SAVR; reduced RV–PA coupling at baseline and 30 days showed strong association with cardiac death at 5 years.

AS, aortic stenosis; HF, heart failure; PA, pulmonary artery; PARTNER, Placement of Aortic Transcatheter Valve; PASP, pulmonary artery systolic pressure; RV, right ventricular; RVLS, right ventricular longitudinal strain; SAVR, surgical aortic valve replacement; TAPSE, tricuspid annular plane systolic excursion; TAVR, transcatheter aortic valve replacement; TTE, transthoracic echocardiography.

^a^ The proportion of patients exhibiting RV-PA uncoupling.

^b^ Measured by RVFWLS/PASP ratio.

Sultan et al. (5) performed a retrospective analysis of 457 patients undergoing TAVR procedure. Based on the TAPSE/PASP ratio, patients were divided into quartiles. The baseline TAPSE/PASP ratio had a linear relationship with 2-year all-cause mortality in TAVR recipients. After multivariable adjustment, RV-PA coupling remained independently associated with outcomes. They concluded that RV-PA coupling has a dose-response relationship with survival and that pre-TAVR TAPSE/PASP evaluation should be incorporated into the shared decision-making process as an integrative approach. Similarly, in a 10-year follow-up study of 56 TAVR candidates with HF and AS, Vizzardi et al. (45) found that RV-PA coupling was a better predictor for risk stratification. They emphasized the role of comprehensive assessment of deformation and RV-PA coupling in predicting long-term outcomes in TAVR candidates.

Recent studies have found that pre-TAVR RV-PA coupling is predictive of cardiovascular death. Meucci et al. (36) studied 900 patients who underwent TAVR at 2 tertiary centers to evaluate the prognostic impact of RV-PA coupling and its association with all-cause mortality. RV-PA coupling was assessed by TAPSE/PASP. More than half of the patients had RV-PA uncoupling before TAVR (58%; severe in 15%). After TAVR, 45% of patients had RV-PA uncoupling and 10% had severe uncoupling. Among all 407 patients with post-TAVR uncoupling, 85% (347) patients had persistent uncoupling, while 15% had new-onset uncoupling. They found that either new-onset or persistent post-TAVR RV-PA uncoupling was associated with higher mortality at a median follow-up of 40 months. In contrast, there was no significant difference in survival between normal pre-TAVR RV-PA coupling and uncoupling. Notably, patients with “recovered coupling” tended to have better outcomes. The reason why the indicator of RV-PA coupling elevated possibly attributed to favorable unloading effects of TAVR on the LV and ultimately a reduction of PASP (46, 47, 51). Parasca et al. (47) used the RVFWLS/PASP ratio as an estimate of RV-PA coupling, and normal coupling was defined as RVFWLS/PASP ≥0.63. 160 patients with severe AS who underwent TTE examinations including STE myocardial deformation analysis of RV function before and one month after TAVR were enrolled (47). They found that the RVFWLS/PASP ratio was a novel non-invasive metric of RV-PA coupling and a predictor of outcome in patients with severe AS undergoing TAVR (47).

Most of the aforementioned investigators emphasized the role of comprehensive assessment of deformation and RV-PA coupling in predicting long-term outcomes in TAVR candidates. Thus, measurement of RV-PA coupling before and after TAVR is helpful in determining appropriate clinical strategies and risk stratification.

#### 3.1.2 Surgical aortic valve replacement

Surgical aortic valve replacement (SAVR) is more commonly used in patients younger than 75 years with low risk (the Society of Thoracic Surgeons predicted risk of mortality score or the European System for Cardiac Operative Risk Evaluation II < 4%) for surgical replacement (33). Compared to TAVR, there are fewer studies focusing on the relationship between RV-PA coupling and SAVR. Cahill et al. (6) conducted a study of 570 patients, 301 underwent TAVR, and 269 underwent SAVR in the PARTNER (Placement of Aortic Transcatheter Valve) 3 trial. Baseline RV-PA uncoupling was defined by TAPSE/PASP, and patients were stratified as “normal” with a ratio greater than 0.55 mm/mmHg, and “uncoupling” with a ratio less than 0.55 mm/mmHg. After 2-year follow-up, RV-PA uncoupling patients had an increased incidence of the endpoint, indicating that it was associated with higher risk of worse outcomes. What's more, RV-PA coupling decreased in patients who underwent SAVR at the early stage, and was sustained for 2 years (6). Another study also found that the TAPSP/PASP ratio was significantly reduced at 30 days and 1 year after SAVR (50). They concluded that reduction in RV-PA coupling is an independent predictor of increased risk of cardiac mortality at 5 years following SAVR (50). Hence, RV-PA coupling may be helpful in guiding treatment in patients undergoing SAVR.

### 3.2 Tricuspid valve

Moderate to severe TR can cause right heart failure (52, 53), and is associated with increased morbidity and mortality (54–56). Secondary TR is more common than primary TR, and is significantly associated with left-sided valvular disease and LV dysfunction (57–59). In clinical practice, TR interventions are usually initiated too late due to other comorbidities (60, 61), and irreversible RV dilatation and even RV failure may occur (62). In a study of 180 patients with moderate or severe secondary TR, researchers found that RV-PA coupling was associated with mortality and hospitalization for HF (30). RV-PA coupling was measured by 3DE using RV forward SV to ESV to avoid underestimation of PASP. The findings showed that RV forward SV/ESV < 0.40 was strongly associated with higher rates of all-cause mortality and hospitalization for HF, and RV-PA coupling could be considered as a marker to detect RV dysfunction and to predict outcomes before conventional RV function parameters fail (30). Fortuni et al. (63) performed a study of 1,149 patients with secondary TR using TAPSE/PASP to estimate RV-PA coupling. Of these, 41% had RV-PA uncoupling with a TAPSE/PASP ratio less than 0.31 mm/mmHg, and during follow-up, these patients tended to have a higher risk of HF syndromes and higher mortality (63). Thus, they found that RV-PA coupling could be used to improve risk stratification in patients with TR.

In patients with severe TR, surgical treatment carries non-negligible risks and morbidity, therefore, transcatheter tricuspid valve replacement (TTVR) has been developed as an alternative treatment option. TTVR has been shown to be feasible with improved outcomes (64–66). After TTVR, regurgitant blood flowing through the tricuspid valve into the right atrium was reduced and additional afterload was introduced, requiring the right ventricle to contract to compensate for the increased afterload burden (67). Thus, evaluation of RV-PA coupling to predict outcome is an ideal method. Recent studies investigating the predictive value of RV-PA coupling in patients undergoing TTVR are shown in Table 2.

**Table 2:** Studies examining the utility of RV-PA coupling among TTVR recipients.

Author, year	Modality	Study population	RV-PA uncoupling cutoff	Follow up	Baseline RV-PA Uncoupling^a^	Main results
Lurz et al. (64)	TTE RHC	243 patients at high surgical risk underwent TTVR from 2 German centers	<0.29 mm/mmHg	330 days (median data)	Not mentioned	TAPSE/PASP ratio is independently associated with event-free survival in patients undergoing separate TTVR
Brener et al. (68)	TTE (all)	444 patients underwent TTVR from 23 international centers	≤0.406 mm/mmHg	1 year	39.9%	TAPSE/PASP ratio is significantly associated with all-cause mortality; RV-PA coupling is a potent predictor within TTVR recipients
Stolz et al. (69)	TTE RHC	502 patients underwent TTVR from 5 European centers	<0.387 mm/mmHg^b^ <0.303 mm/mmHg^c^	2 years	Not mentioned^d^ 35.1%^e^	TAPSE/PASP ratio is significantly associated with all-cause mortality; the ratio assessed by invasive measurement of PASP data is superior to noninvasive one
Fortmeier et al. (67)	TTE RHC	737 patients underwent TTVR from 5 German and Switzerland centers	≤0.617 mm/mmHg	2 years	60.1%	TAPSE/PASP ratio measured by TTE or RHC is associated with survival rates of TTVR recipients; the ratio predicted by XGB algorithm have superior value of refining risk stratification
Sugiura et al. (70)	TTE	206 patients at high surgical risk underwent TTVR from a single German center	Not identified	201 days (median data)	-	TAPSE/PASP ratio is associated with the composite of mortality and HF rehospitalization, the ratio measured invasively is independently related to survival and superior to noninvasive one

HF, heart failure; MR, mitral regurgitation; PA, pulmonary artery; PASP, pulmonary artery systolic pressure; RV, right ventricular; RHC, right heart catheterization; TAPSE, tricuspid annular plane systolic excursion; TTVR, transcatheter tricuspid valve repair; TTE, transthoracic echocardiography; XGB, extreme gradient boosting.

^a^ The proportion of patients exhibiting RV-PA uncoupling.

^b^ Echocardiographically estimated ratio.

^c^ right heart catheterization estimated ratio.

^d^ defined by echocardiographically estimated ratio.

^e^ defined by right heart catheterization estimated ratio.

In 444 patients undergoing TTVR, the degree of TR reduction in TTVR was independently associated with a reduction in post-TTVR RV-PA coupling. Multivariable Cox regression analysis showed that a higher TAPSE/PASP ratio was associated with a lower risk of all-cause mortality (68), confirming RV-PA coupling as a powerful marker for predicting all-cause mortality and helping to select patients suitable for TTVR (68). In a study of 206 patients undergoing TTVR at high surgical risk, the RV-PA coupling ratio was calculated by TAPSE/PASP at baseline (70). PASP was measured echocardiographically or invasively and referred to as ePASP and iPASP respectively. Patients with a lower TAPSE/PASP ratio tended to have a higher risk of death or HF rehospitalization within one year. Additionally, they demonstrated that TAPSE/iPASP (c-statistic 0.695) had a greater predictive value for outcomes compared with TAPSE/ePASP (c-statistic 0.565) (70). Stolz et al. (69) conducted a study of 502 patients who underwent tricuspid valve edge-to-edge repair (TEER) for severe TR. Patients with RV-PA uncoupling had a lower survival rate at 1- and 2-year follow-up, and TAPSE/iPASP was better in predicting mortality (69). In another multicenter study of 737 patients undergoing TTVR for severe TR, RV-PA coupling was defined as the ratio of TAPSE to mean pulmonary artery pressure (mPAP) measured invasively by right heart catheterization (67). In addition, they used an extreme gradient boosting (XGB) algorithm to predict mPAP levels after training on 10 echocardiographic parameters, which could refine risk stratification before TTVR without invasive surgery, and provided the ability to calculate mPAP levels using echocardiographic input parameters (67). Moreover, by multivariate regression analysis, they found that predicted TAPSE/mPAP levels were independently associated with 2-year mortality after TTVR (67). In the end, they revealed that patients with preserved predicted RV-PA coupling, defined by TAPSE/mPAP values greater than 0.617 mm/mmHg, had significantly better survival than patients with reduced RV-PA coupling, and XGB may help to develop treatment strategies to improve survival outcomes in patients with reduced RV-PA coupling (67). Thus, RV-PA coupling plays a key role in risk stratification and patient selection for TTVR.

### 3.3 Mitral valve

Mitral regurgitation (MR) has the highest incidence of valvular heart disease worldwide (33, 71). Untreated moderate to severe MR is responsible for the progressive LV dilatation and dysfunction, leading to reduced quality of life, high rates of morbidity, mortality, and HF hospitalization (72). Depending on the pathogenesis, MR can be of primary or secondary (PMR/SMR) (73, 74). Conventional surgical approach used to be a feasible therapy for patients with symptomatic MR. However, since SMR is often associated with dilated left ventricle with markedly reduced ejection fraction (33), less invasive therapeutic options have emerged as a preferred alternative to surgery (75). Transcatheter mitral valve repair (TMVR) using the MitraClip and the PASCAL edge-to-edge devices is classified in recent guidelines as a treatment that should be considered to improve outcomes in patients with PMR and SMR, especially those at high surgical risk (76, 77). Owing to the improvement in prognosis and quality of life, mitral valve TEER has been performed more frequently in clinical practice (33, 76). Therefore, predicting the outcome after TEER seemed to be more important than ever. In recent years, RV-PA coupling has been proposed as a novel parameter for risk stratification in the TEER population. The relevant studies are listed in Table 3.

**Table 3:** Studies examining the utility of RV-PA coupling among TMVR recipients.

Author, year	Modality	Study population	RV-PA uncoupling cutoff	Follow up	Baseline RV-PA Uncoupling^a^	Main results
Karam et al. (78)	TTE and TEE	817 patients underwent TMVR in 8 European tertiary centers	<0.274 mm/mmHg	2 years	25.8%	TAPSE/PASP ratio is significantly associated with all-cause mortality in TMVR recipients as a continuous variable
Trejo-Velasco et al. (79)	TTE and RHC	228 patients underwent TMVR from 17 Spanish centers	≤0.35 mm/mmHg	1 year	50%	TAPSE/PASP ratio is significantly associated with all-cause mortality, HF readmissions and STS-Score
Brener et al. (80)	TTE	372 patients enrolled in the COAPT trial	<0.5%/mmHg^b^	2 years	70.2%	RVFWLS/RVSP ratio is significantly and independently associated with the composite of all-cause mortality and hospitalization for HF as a continuous variable
Popolo Rubbio et al. (81)	TTE	165 patients treated with the MitraClip from a single Italian center	≤0.36 mm/mmHg	1 year	38.1%	TAPSE/PASP ratio is strongly associated with all-cause mortality and HF hospitalization
Tua et al. (15)	TTE	52 patients treated with the MitralClip from two Italian centrers	<0.14 mm/mmHg^c^	1 year	57.7%	*Δ*(TAPSE/PASP) is not significantly associated with death; TAPSE/PASP is associated with the composite endpoint
Sugiura et al. (70)	TTE	744 patients treated with the MitralClip from 3 German centers	Not identified	18 months (median data)	-	TAPSE/PASP ratio is significantly associated with the composite of all-cause mortality and hospitalization for HF
Adamo et al. (82)	TTE	501 patients underwent TMVR from 13 Italian centrers	<0.36 mm/mmHg	584 days (median data)	51.5%	TAPSE/PASP ratio is significantly associated with long-term all-cause mortality; responders with improvement in TAPSE/PASP ratio is associated with better outcomes
Shechter et al. (83)	TTE	707 patients underwent TMVR from a single Swiss center	<0.37 mm/mmHg	1 year	50%	TAPSE/PASP ratio is not associated with outcomes of primary MR patients undergoing TMVR; TAPSE/PASP ratio is independently associated with excessive postprocedural mortality and HF hospitalizations in functional MR patients
Koschutnik et al. (84)	TTE RHC	156 patients underwent TMVR from a single Austrian center	<0.36 mm/mmHg	2 years	64%	RV-PA uncoupling is significantly associated with a combined endpoint consisting of HF hospitalization and death both in primary and secondary MR

COAPT, Cardiovascular Outcomes Assessment of the MitraClip Percutaneous Therapy for Heart Failure Patients With Functional Mitral Regurgitation; HF, heart failure; MR, mitral regurgitation; PA, pulmonary artery; PASP, pulmonary artery systolic pressure; RV, right ventricular; FWLS, free wall longitudinal strain; RHC, right heart catheterization; RVSP, right ventricular systolic pressure; STS, Society of Thoracic Surgeons; TAPSE, tricuspid annular plane systolic excursion; TEE, transesophageal echocardiography; TMVR, transcatheter mitral valve repair; TTE, transthoracic echocardiography.

^a^ The proportion of patients exhibiting RV-PA uncoupling.

^b^ Calculated by FWLS/RVSP ratio.

^c^ Defined by median Δ(TAPSE/PASP) calculated by subtracting post-procedural values from pre-procedural values.

Recent evidence demonstrates that improvement in RV-PA coupling after TEER is associated with favorable outcomes. In a study of 156 patients with PMR or SMR undergoing TEER, impaired baseline RV–PA coupling was present in 64% of individuals with a TAPSE/PASP ratio <0.36 (84). Patients with reduced RV-PA coupling had a strong association with HF hospitalization and death, as well as a high risk of non-cardiac conditions such as renal dysfunction (84). Using multivariable Cox regression analyses, Koschutnik et al. (84) found that a TAPSE/PASP ratio <0.36 was independently associated with outcomes. Additionally, the authors found that after TEER, RV remodeling and RV-PA coupling may be reversed to some extent in individuals with impaired RV-PA coupling at baseline, which may provide additional prognostic information for poor outcomes (84). A similar message was delivered by Adamo et al., who elegantly demonstrated that SMR patients with lower baseline TAPSE, higher baseline PASP and postprocedural mitral gradient ≥2+ were more likely to improve their TAPSE/PASP ratio after TEER, and may be associated with lower mortality compared with nonresponders with little change in TAPSE/PASP (82). In addition, several studies have focused on the prognostic value of RV-PA coupling, and emphasizing that higher TAPSE/PASP is associated with a favorable post-procedural outcome. Shechter et al. demonstrated that TAPSE/PASP <0.37 mm/mmHg at baseline was associated with worse preprocedural status and immediate postprocedural outcome (83). Thus, a low TAPSE/PASP ratio was strongly associated with higher post-TEER mortality and HF hospitalization in SMR patients (83). In another series of 165 SMR patients with the same TAPSE/PASP cut-off who underwent MitraClip implantation, a baseline RV–PA uncoupling was associated with a higher rate of all-cause mortality (15). Brener et al. (80) performed their subanalysis of the COAPT (Cardiovascular Outcomes Assessment of the MitraClip Percutaneous Therapy for Heart Failure Patients With Functional Mitral Regurgitation) trial using the RVFWLS/PASP ratio, which also demonstrated the prognostic utility of RV-PA coupling. In conclusion, RV-PA coupling may provide further insight into strategy-making and should be accurately assessed when planning a TEER procedure for severe SMR to improve prognosis and subsequently reverse RV remodeling.

## 4 Congenital valvular disease

CHD is the most common congenital disease, including malformations of the cardiac structures and great vessels (85). Nearly twenty percent of newborns with CHD have right ventricular outflow tract obstruction (86), modern treatments for CHD, such as cardiac intervention and surgical repair, significantly prolong survival (87, 88). Along with surgery [such as surgery for tetralogy of Fallot (TOF), pulmonary artery atresia, patent truncus arteriosus, Ross procedure, etc.], new diseases have emerged, including pulmonary valve dysfunction as a short- or long-term outcome (89). Take TOF for instance, after transannular patch repair, most of these patients will ultimately encounter pulmonary regurgitation (PR), pulmonary stenosis and eventually RV dilatation (90). If left untreated, RV failure would occur during long-term following up (91). Thus, pulmonary valve replacement (PVR) is necessary to prevent irreversible RV failure (90, 92). Previous studies have focused on the assessment of RV dimensions and function, whereas the right ventricle is coupled to a highly compliant low-pressure PA system (93). Hence, the right ventricle and PA should be considered as a unit to assess prognosis after PVR. In or after the surgical treatment of TOF, pulmonary valve repair or replacement is often required to address issues such as pulmonary stenosis or PR (94, 95). Studies focusing on RV-PA coupling in repaired TOF have recently attracted more attention, but research on RV-PA coupling in postoperative TOF patients undergoing PVR has rarely been reported. The most relevant publications are listed in Table 4.

**Table 4:** Studies examining the utility of RV-PA coupling with in patients with repaired TOF.

Author, year	Modality	Age (Years), Mean ± SD	Study population	RV-PA uncoupling cutoff	RV-PA Uncoupling^a^	Main results
Egbe et al. (96)	TTE	28 ± 7 (age of patients with repaired TOF and controls)	84 patients with repaired TOF and 84 healthy controls, 45 patients valvular pulmonic stenosis with previous intervention	Not identified	-	Noninvasively measured RVFAC/RVSP and RVFAC/RVSP are correlated with exercise capacity which is significantly associated with cardiovascular mortality and have potential prognostic role in adults with CHD; RV-PA uncoupling may occur in chronic PR with preserved RV systolic function
Sandeep et al. (23)	CMRI	8.52 ± 5.84^b^ 19.88 ± 10.04^c^	135 postoperative TOF patients from a single Chinese center	Ea/Ees > 1	66.7%	Ea/Ees ratio is significantly associated with declined RV function, and possibility of HF
Buddhe et al. (97)	CMRI and TTE	15.9 ± 4.7^d^ 17.0 ± 6.3^e^	248 individuals, 222 with repaired TOF, 26 with repaired TOF- pulmonary atresia from 14 German centers	Ea/Ees > 1	-	Ea/Ees ratio is correlated with NYHA class II symptoms and declined RV function. Ea/Ees ratio is worse in repaired TOF- pulmonary atresia patients
Cheng et al. (98)	TTE	18.6 ± 8.3	60 patients with repaired TOF and 60 healthy controls from a single H.K center	<0.64^f^ <1.19 m^2^s^−1^ cm^−1^^g^	61.7%^f^ 100%^g^	RVFAC/ESA ratio and S′/indexed RVESA ratio are impaired in repaired TOF patients and associated with LV and RV deformation
Vitarelli et al. (99)	TTE, CMR and 3DE	38.1 ± 11.4	24 patients with repaired TOF and 24 healthy controls from an Italian center	0.31%/mmHg^h^ 0.57%/mmHg^i^ 0.86%/mmHg^j^	-	3DE-derived parameters to access RV-PA coupling have high accuracy and are associated with RV dysfunction and clinical prognosis
Kim et al. (100)	CMRI and TTE	21.9 (19.4–27.5)^k^	48 patients with repaired TOF from a single Korean center	Not identified	-	Impaired RV-PA coupling is associated with irreversible and maladaptive RV changes

CMR, cardiac magnetic resonance; Ee, arterial elastance; Ees, End-systolic elastance; HF, heart failure; LV, left ventricular; PA, pulmonary artery; RV, right ventricular; RVAS, right ventricular global area strain; RVESA, right ventricular end-systolic area; RVESV, right ventricular end-systolic volume; RVFLS, right ventricular freewall longitudinal strain; RVSP, right ventricular systolic pressure; S′, peak tricuspid annular s velocity; TOF, tetralogy of fallot; TTE, transthoracic echocardiogram.

^a^ The proportion of patients exhibiting RV-PA uncoupling.

^b^ Age at TOF Repair.

^c^ Age at PVR.

^d^ Patients with repaired TOF.

^e^ Patients with repaired TOF-pulmonary atresia.

^f^ Measured by the RV area change/ESA ratio.

^g^ Measured by RV peak tricuspid annular s velocity/indexed RV ESA ratio.

^h^ Measured by 3DRVAS/RVESV.

^i^ Measured by 3DRVAS/RVSP.

^j^ Measured by 3DRVFLS/RVESA.

^k^ median (interquartile range); 3DE, three-dimensional echocardiography.

In a retrospective study of 135 postoperative TOF patients for PVR, noninvasive measurements by CMR imaging were performed in all patients, and the ratio of ESV/SV equal to Ea/Ees was introduced to define RV-PA coupling (ESV/SV ≤ 1 for coupling and >1 for uncoupling). RV-PA uncoupling was observed in 90 patients and 45 patients had a coupled RV-PA by CMR (23). Patients who underwent PVR had improved RV-PA coupling, supporting the idea that timely PVR can improve RV function. PA system maladapted to chronic RV loading explained the occurrence of uncoupling. Moreover, Sandeep et al. (23) found that Ea/Ees was negatively correlated with RVEF, which represents RV function that is directly correlated with volume overload and pulsatile afterload in the meantime; thus, assessment of RV-PA coupling following TOF repair is a more comprehensive way to reflect the hemodynamic burden of the right ventricle (23). Additionally, Kim SJ and colleagues (100) studied a total of 48 patients with repaired TOF and performed both cardiac catheterization and CMR imaging to assess hemodynamic parameters; 30 patients received PVR and most of them had RV-PA uncoupling. They found that no significant change in RV-PA coupling ratio was found after PVR. By univariate analysis, higher ESV/SV ratio was identified as a risk factor for adverse outcome, and they then concluded that RV-PA coupling is an important predictor for postoperative outcome (100).

As mentioned above, researchers have paid more attention to the RV-PA coupling in repaired TOF without PVR. In a study of 60 patients with repaired TOF, RVFAC/end-systolic area (ESA) ratio and peak tricuspid annular s velocity/indexed RV ESA ratio were assessed to represent RV-PA coupling. They concluded that RV-PA coupling is impaired in rTOF, while demonstrating for the first time that RV and LV mechanics, as well as severity of PR and tricuspid regurgitation (TR) in patients had correlations with RV-PA coupling (98). Buddhe et al. (97) compared RV-PA coupling in repaired standard TOF and those with repaired TOF-pulmonary atresia using the ratio of RV ESV to pulmonary artery SV. RV-PA coupling was impaired in both cohorts, but was worse in repaired TOF- pulmonary atresia. Notably, after regression analysis, they found that RV-PA coupling was associated with New York Heart Association (NYHA) class, and saw the promising future that RV-PA coupling emerge as an indicator of PA compliance and cardiovascular performance (97). Therefore, RV-PA coupling exerts a major impact on risk stratification and prognosis assessment in patients after TOF with or without PVR.

## 5 Limitations

Although RV-PA coupling has been proven to be a strong marker to predict VHD prognosis postoperatively, limitations of which are not negligible. Invasive measurement method using RHC has high sensitivity and accuracy, but it is highly technically demanding, expensive and unpractical to operate bedside (24). Among non-invasive measurement methods, TAPSE/PASP ratio is the most commonly used due to its convenience, however, in case of modest to severe TR, PASP may be underestimated that leads to inaccurate RV-PA coupling measurement. In addition, TAPSE together with S′ is angle dependent. 3D-TTE derived SV/ESV avoids estimation of PASP but have high quality image requirements. Thus, further advancements in non-invasive techniques are in great demand. Another limitation is that the calculated cutoff value of RV-PA coupling via echocardiography varies significantly. It can decrease the importance of its measurement and building prognostic model.

## 6 Summary and prospect

RV-PA coupling has been considered as a strong marker to be implied to multiple conditions, among which is VHD. With the development of technology, treatments for VHD are diversiform and efficient enough to handle conditions that used to be troublesome. However, if unable to distinguish advanced stage of disease progression, advanced treatments to change the disease may be futile. Moreover, some patients may choose conservative treatments, monitoring their daily behavior of various risk conditions are vital to survival. RV-PA coupling has been shown to be a sensitive index for predicting prognosis both in conditions of congenital and acquired valvular diseases. Therefore, it should be routinely assessed as part of the comprehensive echocardiographic or CMR evaluation to help clinicians stratify risk, guide treatment, and improve prognosis. It is vital to develop new method to increase the accuracy, reproducibility, and time efficiency of the assessment of RV-PA coupling in clinical practice. For example, artificial intelligence approach has the potential to automatedly obtain RV-PA coupling and predict poor prognosis in VHD patients.
